# Performance of 2D BLADE turbo gradient- and spin-echo diffusion-weighted imaging in the quantitative diagnosis of recurrent temporal bone cholesteatoma

**DOI:** 10.1186/s12880-022-00860-z

**Published:** 2022-07-27

**Authors:** Mengyan Lin, Yue Geng, Yan Sha, Zhongshuai Zhang, Kun Zhou

**Affiliations:** 1 Shanghai Institute of Medical Imaging, Shanghai, 200032 China; 2 Department of Radiology, Eye & ENT Hospital of Fudan University, 83 Fenyang Road, Shanghai, 200031 China; 3 Scientific Marketing, Siemens Healthcare, Shanghai, 200336 China

**Keywords:** Cholesteatoma, Temporal bone, Magnetic resonance imaging, Diffusion weighted imaging

## Abstract

**Background:**

Diffusion-weighted imaging (DWI) has become an important tool for the detection of cholesteatoma. The purpose of this study was to explore the value of 2D BLADE turbo gradient- and spin-echo imaging (TGSE BLADE) DWI in the quantitative diagnosis of recurrent temporal bone cholesteatoma (CS).

**Methods:**

From March 2018 to October 2021, 67 patients with suspected recurrence of temporal bone CS after assessment by clinical otorhinolaryngologists who had undergone previous ear surgery for CS were prospectively evaluated by magnetic resonance imaging (MRI). Two radiologist assessed images independently. Quantitative parameters such as signal intensity ratio (SIR) calculated using, as a reference, the inferior temporal cortex (SIRT) and the background noise (SIRN), apparent diffusion coefficient (ADC) value, and ADC ratio (with pons as reference) measured on TGSE BLADE sequences were assessed. Using receiver operating characteristic (ROC) curve analysis, the optimal threshold and diagnostic performance for diagnosing recurrent CS were determined. Pair-wise comparison of the ROC curves was performed using the area under the ROC curve (AUC).

**Results:**

Finally, 44 patients were included in this study, including 25 CS and 19 non-cholesteatoma (NCS). Mean SIRT and mean SIRN on TGSE BLADE DWI were significantly higher for CS than NCS lesions (p < 0.001). Meanwhile, mean ADC values and mean ADC ratios on ADC maps were significantly lower in the CS group than in the NCS group (p < 0.001). According to ROC analysis, the diagnostic efficacy of quantitative parameters such as SIRT (AUC = 0.967), SIRN (AUC = 0.979), ADC value (AUC = 1.0), and ADC ratio (AUC = 0.983) was significantly better than that of qualitative DWI (AUC = 0.867; p = 0.007, 0.009, 0.011 and 0.037, respectively).

**Conclusions:**

Residual/recurrent temporal bone CS can be accurately detected using quantitative evaluation of TGSE BLADE DWI.

## Background

Cholesteatoma (CS) is a disease characterized by abnormal growth of keratinized squamous epithelium, which is easy to invade the surrounding structure [1] and needs surgical resection. According to a meta-analysis [2], the postoperative recurrence rate of CS is high, ranging from 9 to 70% depending on the surgical approach. Hence, second look surgery was routinely performed in the past [3]. However, in addition to the general surgical injury, the operation of middle ear CS will also lead to complications such as wax deposition, otorrhea, maze fistula and so on [4].

BLADE, also called PROPELLER, is a turbo spin-echo (TSE)-based diffusion-weighted imaging (DWI) technique. Since the advent of propeller TSE DWI [5], it has been shown to perform well in detecting cholesteatoma [6–8]. 2D BLADE turbo gradient echo and spin echo imaging (TGSE BLADE) is the latest modified diffusion sequence using BLADE technology, and studies have shown that TGSE BLADE is superior to readout-segmented echo-planar imaging (RESOLVE) DWI in detecting cholesteatoma [9, 10]. However, the diagnostic efficacy of TGSE BLADE sequence for recurrent cholesteatoma, as well as its quantitative parameters remain to be further studied.

Signal intensity (SI) on DWI and apparent diffusion coefficient (ADC) on ADC maps are quantitative indicators for detecting CS [11–13]. However, the measurement of SI and ADC values is at least partially influenced by the acquisition technique, the imaging system, and the post-processing platform used [13–15]. Using the SI of subtemporal cortex (SIRT) and background noise (SIRN), and the ADC of the pons (ADC ratio) as references, the calculated quantitative index is more valuable for research [13, 16, 17].

Therefore, the purpose of this study was to explore the value of TGSE BLADE DWI in the quantitative diagnosis of recurrent temporal bone CS.

## Methods

### Patients selection

This study was approved by the Internal Review Board and written consent was obtained from each of the patients. From March 2018 to October 2021, 67 patients with suspected recurrence of temporal bone CS after assessment by clinical otorhinolaryngologists who had undergone previous ear surgery for CS were prospectively evaluated by a dedicated protocol, including T1 weighted image (T1WI), T2 weighted fat-suppression image (T2WI-FS) and TGSE BLADE DWI. Pregnant women and the patients with cardiac pacemaker/metallic implant or claustrophobia were excluded from the study. Moreover, 23 patients in whom recurrent CS was detected by MRI, were excluded since they did not accept surgical treatment or had co-morbidities that made the surgery impossible. Finally, there were 44 patients (24 males and 20 females, age range 13–57 years, mean age 34.2 years) included in this prospective study. They all underwent a second surgery, and the surgical method was wall-down surgery.

### MRI protocol

All patients underwent axial T1WI, T2WI-FS, TGSE BLADE DWI (a prototype TGSE BLADE DWI sequence) at 3 Tesla scanner (MAGNETOM Prisma, Siemens Healthcare, Erlangen, Germany) with a 64-channel brain coil. The parameters for TGSE BLADE DWI were as follows: TR/TE = 4000/62 ms; slice thickness/gap = 2/0.2 mm; slices = 21; bandwidth = 520 Hz/Px; FOV = 280 × 280 mm^2^; matrix = 192 × 192; voxel size = 1.5 × 1.5 × 2.0 mm^3^; NEX = 1; diffusion mode = 4 scan trace; b = 0, 1000 s/mm^2^; turbo factor = 13; EPI factor = 3; and data acquisition time = 3 min 46 s. The readout was performed by Alsop method in this study [18].

### Image assessment

#### Qualitative analysis

For each patient, two observers (9 years and 12 years working experience in head and neck imaging, respectively) assessed images independently. The cases were randomly presented to the reviewers who were blinded to the clinical data. Combined evaluation of TGSE BLADE DWI images and T1WI/T2WI images was performed and the presence of CS was scored: diagnostic confidence scores (1 = very low, 2 = low, 3 = medium, 4 = high, and 5 = very high).

The diagnosis is based on the relative hyperintensity on DWI compared to brain tissue, unless the lesion is hyperintense compared with cerebral white matter on T1WI images, which is highly suggestive of the possibility of cholesterol granuloma [13, 19]. When both readers assigned identical scores to the individually assessed images, the data were collected; when their initial scoring differed, the final score was recorded by consensus. Scores 4 and 5 were considered positive for the prediction of CS.

### Quantitative analysis

The two radiologists manually delineated regions of interest (ROI) of the lesions in DWI and ADC maps, respectively, on a PACS workstation. SI measurements were obtained using an ROI (2 mm^2^) placed within the brightest part of the signal abnormality on the DWI image. The ROI was then copied onto the ADC map to obtain the ADC value. Using the same ROI size, they also obtained the inferior temporal and the background noise to calculate SIRT and SIRN. The background noise ROI was placed far from the skin profile. We calculated using the following formula: SIRT = SI/SI temporal cortex, SIRN = SI/SI noise. Moreover, ADC of the pons at the level of the internal auditory canal was measured with the same ROI size. The ADC ratio was calculated using, as a reference, the pons with the following formula: ADC ratio = ADC value of the lesion/ADC value of the pons.

The SIRT, SIRN, ADC values, and ADC ratios recorded by the two observers were averaged to obtain the final value and defined as the mean SIRT, mean SIRN, mean ADC value, and mean ADC ratio. With T1WI and T2WI-FS guidance, one reader measured the maximum size of the signal abnormality for each patient on TGSE BLADE DWI images.

### Statistical analysis

Agreements between the presence of CS, SI and ADC measurements were evaluated by using Cohen’s kappa and intra-class correlation coefficient (ICC). The histologic findings served as the gold standard for the presence of a CS. Differences in mean SIRT, mean SIRN, mean ADC value, and mean ADC ratio between CS and non-cholesteatoma (NCS) lesions were evaluated using the independent t-test. Using receiver operating characteristic (ROC) curve analysis, the area under the ROC curve (AUC) and 95% confidence interval (95% CI) were calculated: the optimal threshold of diagnosis was determined, and the sensitivity, specificity, and accuracy were also computed to evaluate the diagnostic efficacy of the parameters. Pair-wise comparisons of ROC curves were performed using AUC. The threshold for significance was set at P < 0.05. In addition, SPSS software, version 20, was used to perform statistical analysis, and MedCalc 18.2.1 was used to draw ROC curves.

## Results

Of the 44 patients finally included in the study, the interval between the initial surgery and the second-look MRI was 3–147 months (median, 53 months). The interval between second-look MRI and the secondary operation was 0–142 days, with a mean interval of 46 days. Pathological findings confirmed 25 cases of CS, 19 cases of NCS, including 16 cases of otitis media and 3 cases of cholesterol granuloma. Size of residual/recurrent CS ranged from 2.4 to 26.7 mm.

### Qualitative analysis

**Fig. 1:**
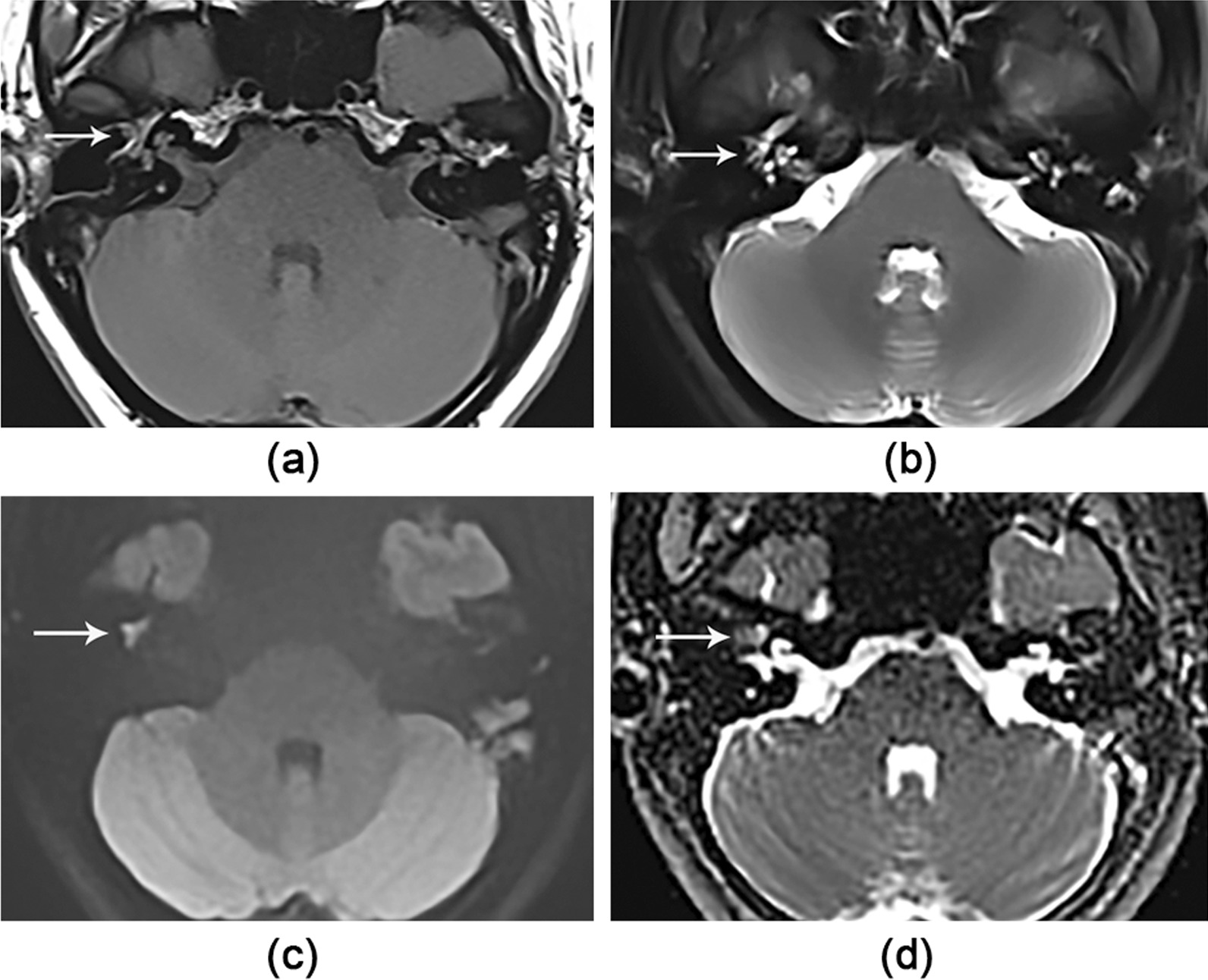
A 22-year-old female patient presented with true-positive CS of 2.4 mm in the right side. Axial images show the lesion (white arrow) as high signal on (**b**) the T2WI-FS, (**c**) TGSE BLADE DWI b1000 image and (**d**) ADC map, and an iso-intensity on (**a**) the T1WI compared to brain tissue

**Fig. 2:**
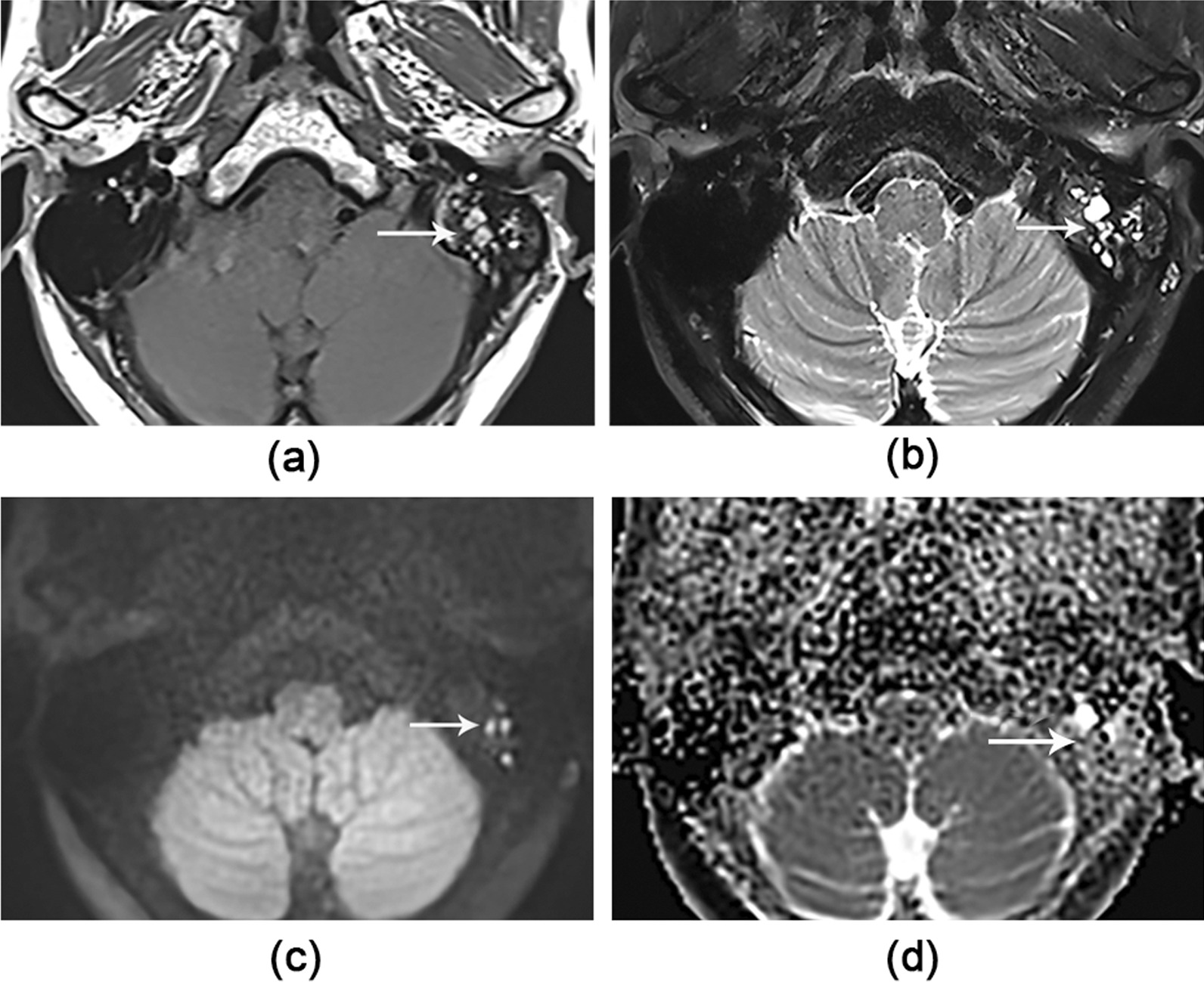
A 52-year-old female patient with false-negative CS in the left side. Axial images show the lesion (white arrow) as high signal on (**a**) first the T1WI, then (**b**) T2WI-FS and (**c**) TGSE BLADE DWI b1000 image, and a low signal on (**d**) the ADC map

In 25 cases of CS, the reviewers found true-positive in 21/25 (84%) (c.f. Fig. 1 for representative example) and false-negative in 4/25 (16%). Three of the four false-negative results were isointense on DWI with infiltration of numerous inflammatory cells and the formation of granulation tissues enclosing a small squamous epithelium area of tissue liner; the other one was associated with T1WI hyperintensity although they presented hyperintensity on DWI (c.f. Fig. 2 for representative example), resulting in suspicious cholesterol granulomas, consistent with high signal intensity on DWI due to T2 shine-through effect of cholesterol granulomas [20].

**Fig. 3:**
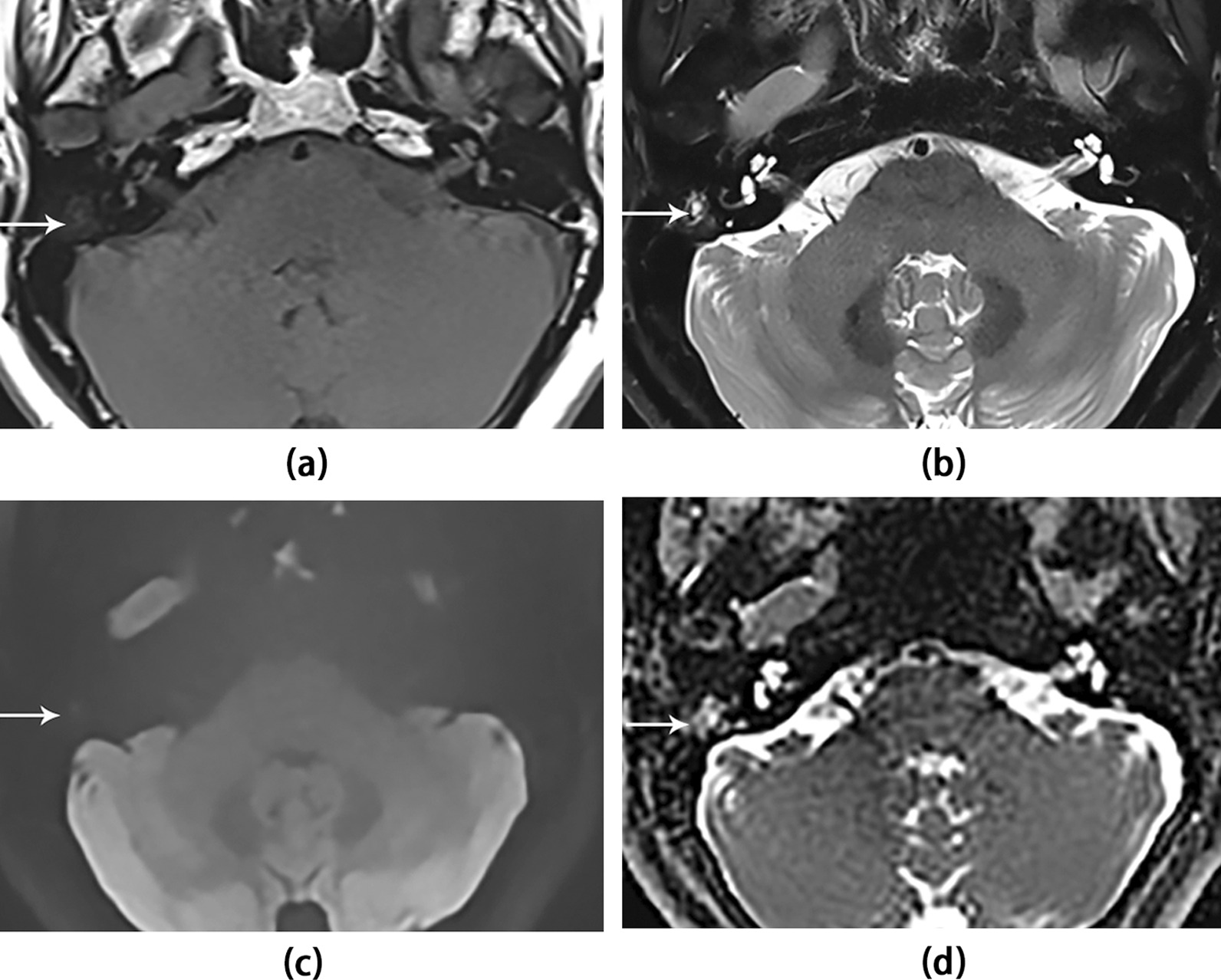
A 41-year-old female patient with true-negative NCS on the right side. Axial images show the lesion (white arrow) as low signal on (**a**) the T1WI and (**c**) TGSE BLADE DWI b1000 image, and high intensity on (**b**) the T2WI-FS and (**d**) ADC map

**Fig. 4:**
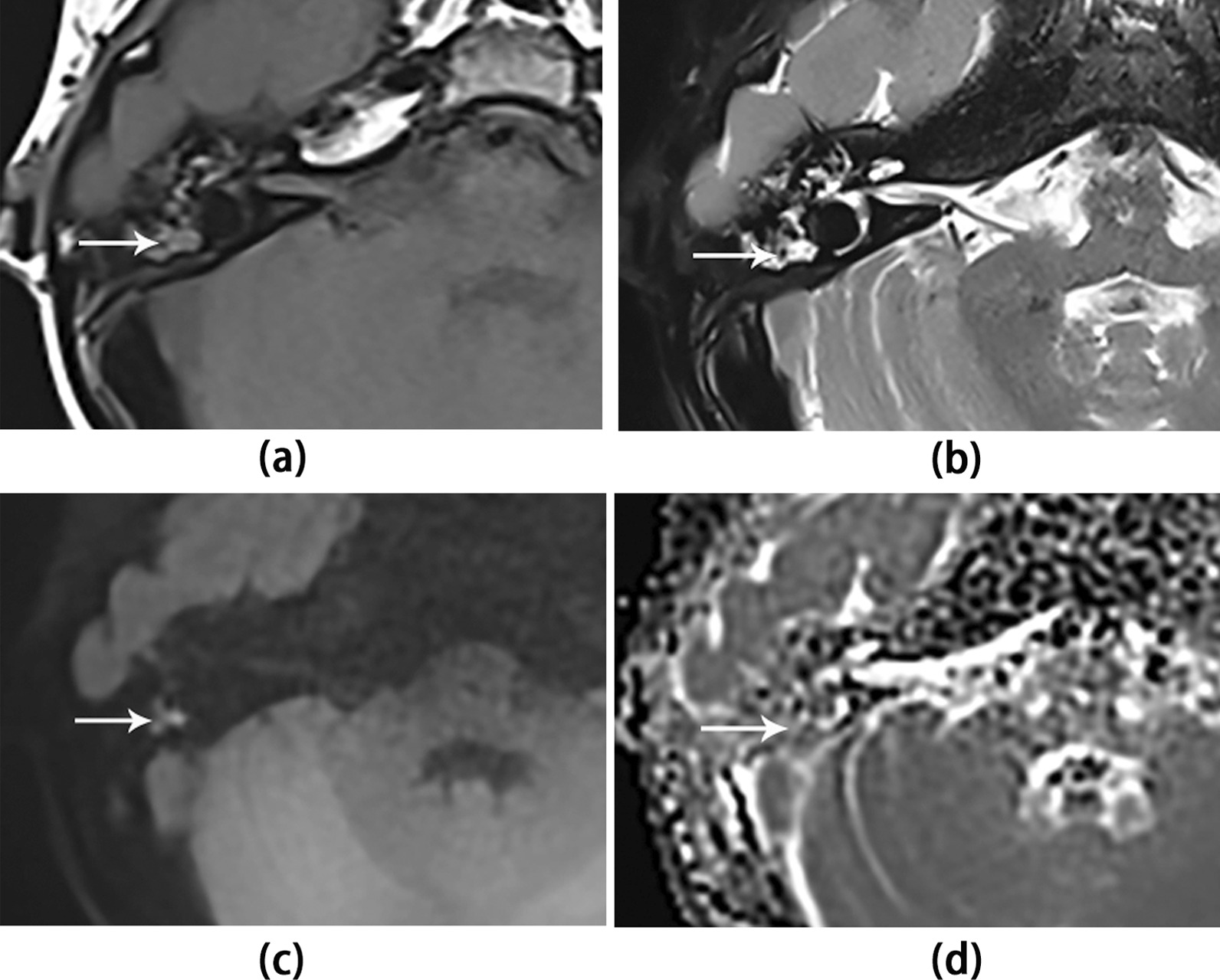
A 37-year-old male patient with false-negative NCS on the right side. Axial images show the lesion (white arrow) as low signal on (**a**) the T1WI and (**d**) ADC map, and high signal on (**b**) the T2WI-FS and (**c**) TGSE BLADE DWI b1000 image

In 19 cases of NCS, the reviewers found true- negative in 17/19 (89.5%) (c.f. Fig. 3 for representative example) and false-positive in 2/19 (10.5%) on DWI, which showed high signal intensity on DWI. The two false-positive results were mastoid abscess and cholesterol crystals (c.f. Fig. 4 for representative example). The diagnostic sensitivity, specificity, and accuracy of qualitative DWI were 84.0%, 89.5%, and 86.4%, respectively (Table 1).

**Table 1:** Sensitivity, specificity, accuracy, and cut-off value of TGSE BLADE DWI parameters for the diagnosis of CS

	Cut-off value	Sensitivity (%)	Specificity (%)	Accuracy (%)
TGSE BLADE DWI		84.0	89.5	86.4
TGSE BLADE SIRT	> 0.9	88.0	94.7	90.0
TGSE BLADE SIRN	> 2.4	92.0	94.7	93.2
TGSE BLADE ADC value	< 1.3	100	100	100
TGSE BLADE ADC ratio	≤ 2.0	92.0	100	95.5

ADC values are presented as 10^−3^mm^2^/s

### Quantitative analysis

**Fig. 5:**
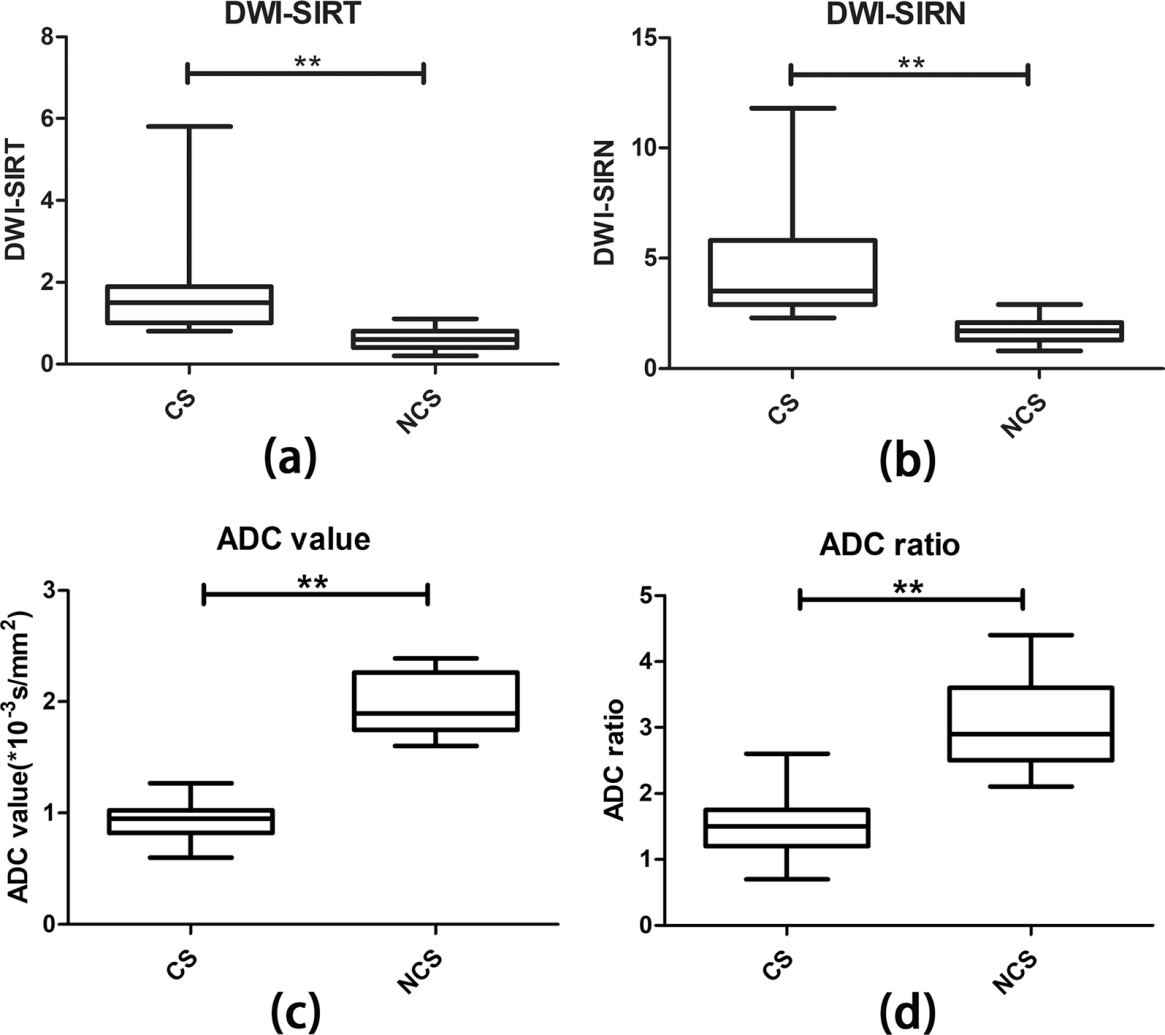
Comparison of mean SIRT (**a**), SIRN (**b**), ADC value (**c**) and ADC ratio (**d**) in CS and NCS patients. Boxplots showing the distribution of SIRT, SIRN, ADC values and ADC ratio. Center lines show the medians; box limits indicate the 25th and 75th percentiles; whiskers extend to minimum and maximum values. **p < 0.001

**Fig. 6:**
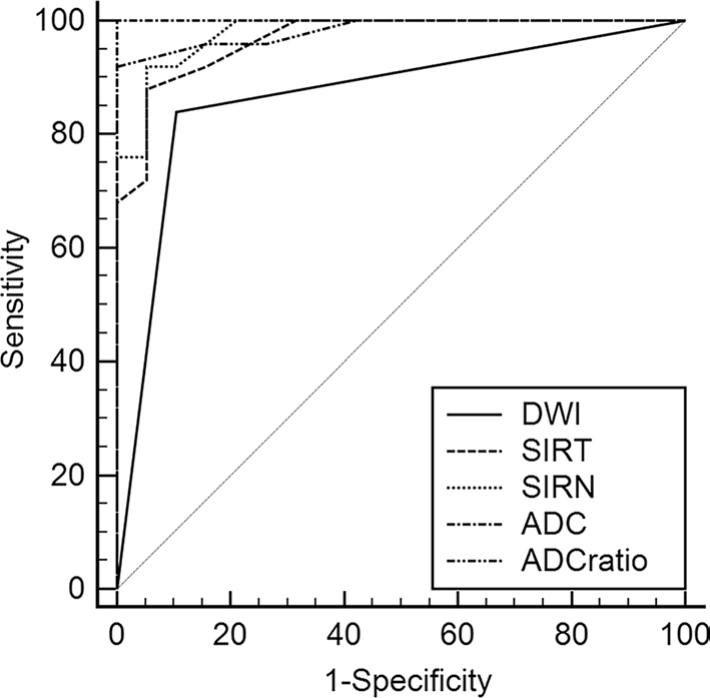
Comparison of diagnostic performance between qualitative DWI and quantitative parameters including mean SIRT, mean SIRN, mean ADC value, and mean ADC ratio (ROC curve analysis)

The mean SIRT and mean SIRN were notably higher in CS than in NCS lesions (p < 0.001). And the mean ADC values and mean ADC ratios were significantly lower in the CS group than in the NCS group (p < 0.001; Table 2). The optimal threshold and the sensitivity, specificity, and accuracy of parameters for CS diagnosis are show in Table 1. The comparison of the quantitative indices in CS and NCS patients are illustrated with Boxplots in Fig. 5. The ROC curves and AUCs on the diagnostic performance of TGSE BLADE parameters are shown in Fig. 6 and Table 3. The AUCs of SIRT (AUC = 0.967), SIRN (AUC = 0.979), ADC value (AUC = 1.0), and ADC ratio (AUC = 0.983) were significantly higher than those of qualitative DWI (AUC = 0.867; p = 0.007, 0.009, 0.011 and 0.037, respectively). There was excellent agreement between the two reviewers (Table 4).

**Table 2:** Mean values of SIRT, SIRN, ADC value and ADC ratio in CS and NCS

	CS (n = 25)	NCS (n = 19)	p-value
TGSE BLADE SIRT	1.6 ± 1.0 (0.8–5.8)	0.6 ± 0.2 (0.2–1.1)	< 0.001
TGSE BLADE SIRN	4.3 ± 2.2 (2.3–11.8)	1.7 ± 0.5 (0.8–2.9)	< 0.001
TGSE BLADE ADC value	0.9 ± 0.2 (0.6–1.3)	1.9 ± 0.3 (1.6–2.4)	< 0.001
TGSE BLADE ADC ratio	1.5 ± 0.4 (0.7–2.6)	3.0 ± 0.7 (2.1–4.4)	< 0.001

The values are given as mean ± standard deviation (range); ADC values are presented as 10^−3^mm^2^/s

**Table 3:** Comparison of AUCs of TGSE BLADE parameters

	AUC (95% CI)	p-value
TGSE BLADE DWI vs TGSE BLADE SIRT	0.867 (0.731–0.951) vs 0.967 (0.864–0.998)	0.007
TGSE BLADE DWI vs TGSE BLADE SIRN	0.867 (0.731–0.951) vs 0.979 (0.882–1.0)	0.009
TGSE BLADE DWI vs TGSE BLADE ADC value	0.867 (0.731–0.951) vs 1.0 (0.920–1.0)	0.011
TGSE BLADE DWI vs TGSE BLADE ADC ratio	0.867 (0.731–0.951) vs 0.983 (0.889–1.0)	0.039

**Table 4:** Interobserver agreement values for TGSE BLADE DWI parameters

	k	ICC	p-value
TGSE BLADE DWI	0.873	–	< 0.001
TGSE BLADE SIRT	–	0.963	< 0.001
TGSE BLADE SIRN	–	0.945	< 0.001
TGSE BLADE ADC value	–	0.933	< 0.001
TGSE BLADE ADC ratio	–	0.744	< 0.001

## Discussion

The results of this study indicate that the quantitative parameters of TGSE BLADE DWI are helpful in distinguishing residual/recurrent temporal bone CS from NCS lesions.

TGSE BLADE is a multi-shot technique that solves the phase instability inherent in multi-shot DWI by acquiring k-space centers each time and using these data for phase correction. For each shot, a rotating rectangular part of k-space was acquired, after which the reading within the rectangle was performed using the TGSE method (a method of combining TSE and gradient-echo). In contrast to prior BLADE-based methods, TGSE BLADE uses gradient echoes to reduce the acquisition time and minimizes the susceptibility artifacts by separating the gradient echoes and spin echoes between diffusion preparation and data acquisition [9]. Unlike the single diffusion encoding direction of half-Fourier acquisition single-shot turbo spin-echo (HASTE), TGSE BLADE provides multidirectionally dispersed diffusion patterns, such as 3-scan-trace, 4-scan-trace, etc., to reduce the direction dependency of signal intensities [21].

The diagnostic efficacy of TGSE BLADE DWI in residual/recurrent temporal bone cholesteatoma has not been previously reported, and it belongs to the non-echo planar imaging (non-EPI) techniques. In this study, the diagnostic sensitivity, specificity, and accuracy of qualitative DWI were 84%, 89.5%, and 86.4%, respectively. Pooled sensitivity of non-EPI DWI for the detection of residual/recurrent CS in a recent meta-analysis was 80–82% with a specificity of 90–100% [22], which is generally consistent with the results of this study. In addition, it has been shown that the RESOLVE sequence has a sensitivity of 68–76% and a specificity of 60% in primary cholesteatoma [23]; the sensitivity and specificity of TGSE BLADE DWI in this study were better than those of this sequence, which is consistent with the results of previous studies [10].

For cholesteatoma imaging, it has been proposed to use the pons, infratemporal cortex and background noise as a reference [12, 13, 24]. In this study, quantitative parameters such as SIRT, SIRN, ADC value, and ADC ratio have excellent ability to differentiate CS from NCS. These findings are consistently supported by previously reported results [12, 25, 26]. Recently, Cavaliere et al. [27] reported that in 109 patients, the ADC value of non-EPI yielded 97% sensitivity and 100% specificity, yet the size of the CS was not mentioned. And it has been shown [13] that the diagnostic sensitivity, specificity and accuracy of SIRT are 100%. These conclusions, like the present results, reflect the superiority of using quantitative parameters for the diagnosis of CS. In this study, the parameter has good sensitivity and specificity for distinguishing CS from NCS lesions when using ADC of 1.27 × 10^−3^ mm^2^/s as the cut-off value, which is basically consistent with the ADC cut-off value of 1.24 × 10^−3^ mm^2^/s calculated by Özgen et al. [13] and the cut-off value of 1.3 × 10^−3^ mm^2^/s calculated by Lingam et al. [11].

The AUCs calculation of ROC curves further showed that quantitative parameters such as SIRT, SIRN, ADC value and ADC ratio had dramatically better diagnostic efficacy compared with qualitative DWI (p = 0.007, 0.009, 0.011 and 0.037, respectively). As in previous studies [11], ADC value was similarly found to be effective in improving the already high specificity of the qualitative analysis. Few studies have evaluated SI values on DWI due to magnetic field inhomogeneities on MRI and intensity variations of scanner-related artifacts, but the calculation of signal intensity ratio (SIR) may improve the reproducibility of numerical measurements to some extent (measurements were highly consistent between the two radiologists). However, specific thresholds for different centers remain to be further assessed.

The diagnostic superiority of TGSE BLADE DWI for small CS [9] was also reflected in the detection of residual/recurrent temporal bone CS, and the smallest diameter CS in this study was 2.4 mm, which was clearly visualized on DWI images (Fig. 1). This is consistent with previous findings [10, 28].

The present study has some limitations. First, only patients with suspected CS recurrence in the temporal bone were included in this study, and not all patients underwent a second surgical exploration after primary ear surgery, so there may be a selection bias. Secondly, the number of cases included in this study was small, which hinders us from concluding that the detection of CS using DWI quantitative parameters may be the first choice after surgery. Finally, the values of the quantitative parameters of the lesion were correlated with the ROI delineated by the observers, so measurement bias was inevitable.

## Conclusion

Residual/recurrent temporal bone CS can be accurately detected using quantitative evaluation of TGSE BLAD DWI. Compared with qualitative DWI, quantitative parameters such as SIRT, SIRN, ADC value and ADC ratio had notably better diagnostic efficacy.

## Data Availability

The datasets used and analyzed in the current study are available from the corresponding author on reasonable request.

## Abbreviations

TGSE BLADE: 2D BLADE turbo gradient- and spin-echo imaging
DWI: Diffusion-weighted imaging
MRI: Magnetic resonance imaging
SI: Signal intensity
SIR: Signal intensity ratio
ADC: Apparent diffusion coefficient
ROI: Region of interest
AUC: Area under the ROC curve
ICC: Intraclass correlation coefficient
